# Improving Self-Control: The Influence of Role Models on Intertemporal Choices

**DOI:** 10.3389/fpsyg.2019.01722

**Published:** 2019-08-02

**Authors:** Gayannée Kedia, Hilmar Brohmer, Marc Scholten, Katja Corcoran

**Affiliations:** ^1^Social Psychology, University of Graz, Graz, Austria; ^2^Department of Marketing, Universidade Europeia, Lisbon, Portugal; ^3^Biotechmed, Graz, Austria

**Keywords:** intertemporal choices, modeling, self-control, observational learning, procrastination, reward, smoking, obesity

## Abstract

The ability to delay rewards is one of the most useful qualities one may wish to develop. People who possess this quality achieve more successful careers, display better interpersonal skills and are less vulnerable to psychopathology, obesity or addictions. In the present online studies, we investigated the extent to which delay-of-reward behaviors in female participants can be improved by observing others mastering it. We developed an intertemporal choice (IC) paradigm in which participants had to make fictitious choices between sooner smaller rewards and later bigger ones (e.g., $150 in 1 week vs. $170 in 4 weeks). In Study 1 (*N* = 186), we found that participants who delayed more had higher socioeconomic statuses and were less likely to procrastinate, smoke or develop obesity. In Study 2 (*N* = 178), we exposed female participants to a role model who, faced with ICs, chose most of the time the delayed option. Results showed that, although participants were only asked to memorize the model’s decisions, they tended to choose the delayed option more often after than before exposure to the model. In Study 3 (*N* = 148), we found that the direction of the influence depended on the model’s behavior: our female participants delayed more after having seen a high delay than a low delay model. In Study 4 (*N* = 370), we confirmed the effect of modeling on ICs in comparison to a control condition and showed that this effect was still significant 3 months after exposure to the model. Altogether, these results speak in favor of a high efficacy of modeling to develop self-control in women.

## Introduction

Among all the qualities one may wish to develop, the ability to control one’s behavior when exposed to temptations is probably the most useful one. Psychological research has demonstrated that people who are able in such situations to delay rewards obtain better academic grades, encounter greater career successes and reach a higher socioeconomic status (SES; Wolfe and Johnson, 1995; Moffitt et al., 2011; Daly et al., 2015; Ishii, 2015). The ability to defer rewards is also related to better social skills, less violent behaviors, healthier habits as well as a lower risk to develop psychopathology, obesity or addictions (Tangney et al., 2004; Reynolds, 2006; Rounds et al., 2007; Appelhans et al., 2016; Grabski et al., 2016). Promoting delay-of-reward behaviors is thus a crucial challenge for parents, educators, politicians and individual persons who would like to overcome self-control failures (Mischel, 2014; Friese et al., 2017). Social learning theory suggests that observing others who master delay-of-reward skills represents an efficient strategy for this purpose (Bandura, 1986).

Indeed, humans often learn by watching others and then imitating, or *modeling*, what they do or say. Modeling can take place in the presence of a live model demonstrating or acting out the behavior or it can rely on symbolic models, such as characters displayed in books, films, television programs, or online medias (Bandura et al., 1961). Learning by observing others considerably reduces the costs of individual learning. Instead of engaging in a laborious and potentially dangerous search by trials and errors of the optimal strategy to solve a problem or accomplish a task, an individual can just observe others successfully mastering this task and reproduce their behavior. Moreover, the ease with which humans identify with others and act alike endows them with the ability to quickly fathom and adopt the norms and behaviors characteristic of their social groups, an essential skill for social integration (Bandura, 1986).

Several articles report that children and teenagers’ ability to delay rewards can be modified through exposure to models (Bandura and Mischel, 1965; Staub, 1972; Stumphauzer, 1972). These studies investigated delay-of-rewards behaviors with an intertemporal choice (IC) paradigm: participants were asked to choose between pairs of rewards differing in both their magnitudes and time of delivery (e.g., $25 today vs. $35 in a week). They were tested before and after having observed an adult make decisions that were counter to their own IC pattern (e.g., low delay children were exposed to a high delay model, whereas high delay children were exposed to a low delay model). Results showed that children and teenagers — whether high or low in delay — adapted their choices to the model’s.

Do these results replicate in adults? Adults tend to be less sensitive to modeling influences than children. In sports, for example, research has shown that reliance on observational learning to improve one’s skills, strategy and performance decreases with age (Law and Hall, 2009). Recent studies have thus tested modeling effect on ICs in adults (Nicolle et al., 2012; Gilman et al., 2014; Garvert et al., 2015; Moutoussis et al., 2016; Calluso et al., 2017; Devaine and Daunizeau, 2017). These studies required participants to observe the ICs made by another person or to predict what this person would choose. Immediately after having observed or predicted the model’s decision, participants had to decide for themselves. Results reported a modeling effect on the participants’ behavior: low delay participants made more delayed ICs after having been exposed to a high delay model (Nicolle et al., 2012; Gilman et al., 2014; Calluso et al., 2017). Moreover, these paradigms were designed to calculate the participant and the model’s delay discounting functions, i.e., the mathematical functions best describing the decline of a reward value with delay to its receipt (Odum, 2011). Some of these studies, although not all of them, reported a transfer of the discounting function: participants’ discounting function shifted toward the model’s after having observed him/her (Garvert et al., 2015; Calluso et al., 2017; Devaine and Daunizeau, 2017).

Previous studies investigating the effects of modeling on adults have important limitations. Firstly, they lack ecological validity. Calculating delay discounting functions requires dozens or even hundreds of trials in order to reach a satisfying statistical power. These paradigms thus involve an intensive exposure that is both exhaustive and remote from everyday life situations. Secondly, the goal of these studies was obvious to their participants. The typical paradigm on which these studies were based had participants observe the model’s decision to an IC and immediately afterward decide for themselves. One could thus argue that the modeling effects observed in these studies were only due to experimental demand effect (i.e., participants conforming their behavior to what they believe is expected from them). The studies reported in the present article were designed to address these limitations.

**FIGURE 1:**
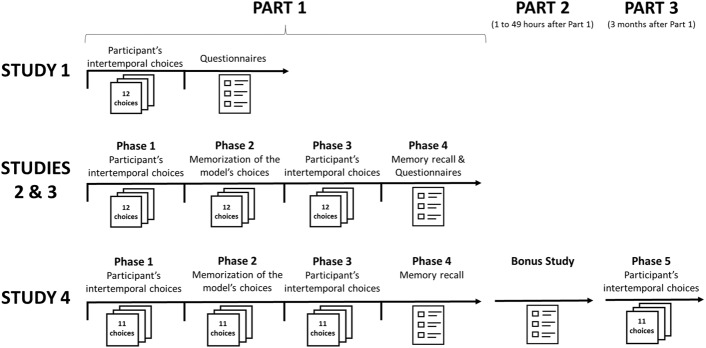
Procedure of the studies.

To test whether a subtler influence would also reveal modeling influences in female adults, we created a short paradigm in which participants’ IC tendency was tested before and after they observed decisions supposedly made by another participant (see Figure 1). Our goal was to investigate participants’ decisions and not their delay discounting function. We only used a dozen of trials per phase and we concealed the goal of our research with a cover story. We presented the survey as an investigation into decision-making and memory and insisted on the importance for participants to make their own decisions. We implemented this paradigm in a series of online studies performed on Amazon’s Mechanical Turk (MTurk), an internet platform that allows people all over the United States to complete online tasks in exchange of a small monetary compensation. Our participants performed the studies in their own environment and they did not have any direct contact with the role model or the experimenter; the psychological pressure to conform to the model’s behavior was thus low (for research on the effects of physical distance on social influence see Haslam et al., 2014).

We ran three online studies aimed at investigating whether ICs are influenced by modeling. In Study 2, we investigated whether people delay more after having been exposed to a high delay model and we manipulated the characteristics of this model to make her more or less inspiring. In Study 3, we tested whether modeling effects were also observable with low delay models. Finally, in Study 4, we investigated whether modeling effects persist over time and the extent to which they can generalize to a new situation or task. In these three studies, we decided to focus on female participants because women are supposed to be more sensitive to social influences than men (Cacioppo and Petty, 1980). Our manipulation being subtler than those of previous studies, we decided to start by testing this effect in a population more likely to display it.

The unfolding of these studies is systematic and unassuming. Our approach is driven by the motivation to replicate previous research and steadily expand these results (for articles promoting this kind of approach, see Asendorpf et al., 2013; Funder et al., 2014; Munafò et al., 2017). We thus begin by demonstrating the validity of our IC paradigm. Psychologists have indeed developed several paradigms to measure a person’s IC tendency and these different paradigms can lead to different results (Matta et al., 2012; Gilman et al., 2014; Calluso et al., 2017). For the reasons mentioned above, in the present project, we decided to rely on an IC paradigm shorter than in most studies. Still, we considered essential for the practical relevance of this research to show that participants’ responses to this task were related to their everyday life behavior. Thus, in Study 1, we investigated the extent to which the number of ICs made in our paradigm correlated with several personality traits, health and socio-economic indicators known to require delaying rewards (Tangney et al., 2004).

In a spirit of transparency, we have made all data (raw and filtered), experimental material and syntaxes accessible on the open science framework and we have preregistered Study 4^1^. We have determined the sample sizes before any data analysis using power analyses. Moreover, we have taken the side of reporting in the current manuscript the studies collected in preparation of this article, whether their results were significant or not. For each of these studies, we report all measures, manipulations and exclusions. For the sake of concision, we report in the main article the main methods and results but more detailed information concerning all studies and complementary results can be found in the Supplementary Material (please note that Supplementary analyses point to the same results as those reported in the main text).

## Study 1

The aim of Study 1 was to validate the IC task that we have developed for this project. To this end, we investigated the correlation between the number of delayed options participants selected in our IC task and several self-reported variables known to be associated with the ability to delay reward (Tangney et al., 2004). We asked participants to report their tendency to procrastinate, their education level, their socioeconomic status, their proneness to experience social anxiety as well as their smoking behavior, their height and their weight [to calculate their body mass index (BMI)].

### Methods

This study, as well as all the other studies presented in this article, was approved by the ethics committee of the University of Graz.

#### Procedure

We programmed this study, as well as the other studies reported in this article, in Questback Unipark (2017). This online study was framed as a decision-making task. We used hypothetical rewards. Previous research reports that hypothetical rewards are similarly discounted as real rewards (Madden et al., 2003; Locey et al., 2011) but other research suggest that this may also depend on the details of the procedure (Frederick et al., 2002; Cohen et al., 2019). It was thus important to demonstrate in this first study that the paradigm we planned to use in the modeling studies was valid.

After filling out the consent form, participants were asked to make 12 hypothetical monetary ICs (e.g., $500 in 1 week vs. $550 in 2 weeks). Delays and amounts were adapted from Scholten et al.’s (2014) Study 2; the offers can be found Supplementary Material. Then, participants had to answer several questions, scales and demographical information. Participants were eventually thanked and offered a debriefing via email. Figure 1 represents the procedure of this study and the three other ones reported in this article.

#### Questionnaires

##### Pure Procrastination Scale

Procrastination refers to the act of putting off or delaying a task that requires immediate attention. Procrastination involves delaying reward to the extent that immediate rewards (e.g., watching TV) receive higher preference than long-term goals (e.g., exercising). The Pure Procrastination Scale includes 12 items that measure a dysfunctional tendency to delay making decisions (e.g., “I waste a lot of time on trivial matters before getting to the final decisions”) and performing tasks (e.g., “I’m continually saying ‘I’ll do it tomorrow”’). Participants evaluate the items on 5-point scales ranging from 1: “very seldom or not true of me” to 5: “very often true of me.” Previous research has demonstrated good psychometrical properties of the scale (Steel, 2010). The data collected in Study 1 sample revealed excellent internal consistency (Cronbach’s α = 0.97).

##### Social Interaction Anxiety Scale (SIAS, Mattick and Clarke, 1998)

Previous research has shown that individuals with social anxiety disorder display increased discounting rates in IC paradigms (Rounds et al., 2007). This may explain their difficulty in social situations to overcome immediate anxiety symptoms (e.g., blushing before speaking) in order to establish potentially pleasant social contacts. The SIAS is composed of 20 items (e.g., “I have difficulty making eye contact with others”) assessed on a 5-point scale ranging from 0: “not at all characteristic or true of me” to 4: “extremely characteristic or true of me.” In our sample of participants, the scale displayed excellent internal consistency (Cronbach’s α = 0.96).

##### Smoking behavior

Dysfunctional delay-of-reward behavior is assumed to play a central role in addictions such as cigarette smoking (Fields et al., 2009; Ishii, 2015). To test whether our IC task replicated this effect, we asked participants (1) whether they smoked (*Yes*, *Occasionally*, *No*), and if so (2) how many cigarettes per day approximately, (3) for how long they have been smoking (in years and months) and (4) the extent to which they have in the past unsuccessfully tried to quit. To assess this *smoking procrastination* behavior, we created 3 items (“Oftentimes I wanted to stop smoking but didn’t manage to do so,” “It happened more than once that I planned to quit smoking or smoke less without success,” “In several attempts to quit smoking or smoke less I failed”) that participants had to rate on a 5 point-scale ranging from 1: “not at all characteristic or true of me” to 5: “extremely characteristic or true of me.” This scale displayed excellent internal consistency (Cronbach’s α = 0.96).

##### Body mass index

Previous research has shown that obese patients delay rewards less than control healthy subjects do (Fields et al., 2011; Thamotharan et al., 2016). Therefore, we asked our participants to provide their height (in feet and inches) and weight (in lbs) and we calculated their BMI (i.e., weight in kilograms over the square of height in meters). According to the United States Department of Health and Human Services, a person with a BMI below 18.5 is considered underweight, a BMI between 18.5 and 24.9 is healthy, between 25 and 29.9 overweight, while a BMI above 30 is obese.

##### Subjective socioeconomic status (SES)

To measure subjective socioeconomic status (SES), participants were asked to place themselves or their family on a 10-rung ladder depicting hierarchy in their society (1: lowest rung corresponding to people who are the worst off, 10: highest rung corresponding to people who are the best off, adapted from Adler et al., 1994).

##### Objective socioeconomic status

We used participants’ yearly income as an index of objective SES. Participants were provided several possible answers and were asked to indicate which category best described the total combined family income for their household in the past 12 months before tax. The response categories were: <$25 000, $25 000 ≤ income < $50 000, $50 000 ≤ income < $75 000, $75 000 ≤ income < $100 000, $100 000 ≤ income < $150 000, ≥$150 000, Don’t know / Not sure, I decline to respond.

In addition, participants had to indicate the number of people currently living in their household, including themselves, and the number of underage children. Finally, we asked them to provide their education level among 6 categories (school, high school, college, bachelor, master, PhD).

##### Demographics

Participants were also asked to report their age, gender, nationality, country of residency, and mother tongue. Study 1 involved many questionnaires; therefore we made it a prerequisite for participants to be native English speakers.

##### Control variables

To make sure that participants were naïve to our study and concentrated on the task, we asked them to indicate whether they already had participated in a similar study (*yes*/*no*), whether they were distracted during the study (“very distracted,” “fairly distracted,” “slightly distracted,” and “not distracted”), and whether they thought we should use their data in our analyses (*yes*/*no*). We used these control variables to select our sample and excluded participants who already had taken part in a similar study, who indicated having been very or fairly distracted while doing the study, and who recommended not to use their data in our analyses. These exclusion criteria were applied in all the studies reported in this article.

#### Participants

Data were collected on Amazon’s Mechanical Turk (MTurk), an internet platform that allows people to complete online tasks. Participants received $1.50 settlement for completing the survey, which lasted about 15 min. We calculated with G^∗^Power (Faul et al., 2007) that, for bivariate correlations, we would need a sample of *N* = 193 (two-tailed) with α = 0.05, 1-β = 0.80 to detect small to medium effects (0.20 < *r* < 0.25).

We collected the data from 226 MTurk workers but only 201 completed it until the end. Among them, 15 were excluded because they reported having been distracted, that their data were not usable or that English was not their mother tongue. Our final sample included 186 participants (85 women, *M*_age_ = 36.53, *SD* = 10.73, sensitivity: *r* = 0.204).

### Results and Discussion

We found that the number of delayed choices made by the participants significantly correlated with their procrastination score (*r*(184) = −0.17, *p* = 0.03), their household income (*r*(184) = 0.25, *p* = 0.001), and their subjective SES (*r*(184) = 0.15, *p* = 0.04). We did not observe any significant correlation with the social anxiety score (*r*(184) = −0.01, *p* = 0.88). In addition, participants who reported smoking chose fewer delayed options than occasional smokers and non-smokers (*F*(2, 183) = 3.99, *p* = 0.02, η*p*^2^ = 0.04) and the more cigarettes participants smoked the lower the number of chosen delayed options (full sample: *r*(184) = −0.18, *p* = 0.02; smokers: *r*(47) = −0.42, *p* = 0.003).^2^ Results of the smoking procrastination items did not indicate any significant correlation with the number of chosen delayed options (*r*(27) = −0.15, *p* = 0.43). However, this latter correlation was performed in a small sample (*N* = 29 smokers) and should thus be interpreted with caution.

Finally, obese participants displayed a marginally significant tendency to delay less than participants with a healthy weight (*t*(132) = 1.69, *p* = 0.09, *d* = 0.307). Descriptive statistics and complementary analyses can be found in the Supplementary Material.

The numerous significant correlations found in this study suggest that the IC task we created for this research captures an important aspect of self-control for everyday life behaviors. This task seems thus to constitute a relevant paradigm to investigate whether delay-of-reward behaviors can be changed by the observation of a model.

## Study 2

The goal of the second study was to test whether participants’ ICs can be influenced by the mere observation of another participant. The model in this study behaved like a *high delayer*, a person who mainly chose the later larger reward. In addition to assessing participants’ delaying style before and after model exposure, we varied the inspiration potential of the model. Previous literature has shown that for a model to be influential, he or she must be perceived as sympathetic, similar and competent for the task at hand (Bandura, 1986). Therefore, we manipulated these characteristics in the description of the model to create *inspiring* and *uninspiring model* conditions.

### Methods

#### Procedure

We invited female MTurk workers to take part in a research on decision-making and memory composed of 4 phases (see Figure 1).

In Phase 1, participants were asked to make 12 hypothetical monetary ICs; the offers and instructions were the same as in Study 1. In Phase 2, they were told to memorize decisions made by their experimental partner, a supposedly other female participant who also had made 12 monetary ICs.^3^ In fact, these decisions were predetermined: 10 times out of 12 the model chose the later larger reward. The pseudo experimental partner or model in this study was introduced in one of two ways. In the *inspiring model* condition, participants could choose their experimental partner from a list of female names. We assumed that participants would select a name that they liked and that this would foster modeling effects. Then, they received a friendly message from her in which she described herself as competent in financial decision-making. The inspiring model was introduced as 1 year older than the participant and had the same nationality and level of education. Conversely, participants in the *uninspiring model* condition were automatically assigned to an experimental partner who was 1 year younger than they were, had a low education level (i.e., high school) and a foreign nationality (i.e., Russian). The uninspiring model introduced herself with poor English and in an unfriendly manner as someone who usually makes bad financial decisions.

In Phase 3, we assessed participants’ ICs after exposure to the model using a decision task encompassing 12 IC offers similar to those of Phase 1. In Phase 4, we asked participants to complete a short memory task implemented to be consistent with the announced purpose of the study. Finally, we asked participants to rate with three items per dimension how similar to the model they felt, how likeable they thought she was, how competent they assumed she was and how often they thought about her during Phase 3 (modeling scale). All IC offers, the details of the experimental manipulation as well as the description of the manipulation check and scales can be found in the Supplementary Material.

#### Participants

This study relied on a 2 (within-subject: pre vs. post-exposure to the model) × 2 (between-subject: inspiring vs. uninspiring model) experimental design. We aimed for small to medium effect sizes as is usually the case in social psychology studies. With 160 participants in a mixed ANOVA with two measures (*r* = 0.50) and two groups (*n* = 80, two- tailed, α = 0.05, 1−β = 0.80), we could expect to find a small population effect (*r*∼0.11).

Participants received $1.50 settlement for completing the survey, which lasted about 15 min. A total of 823 participants clicked on the online survey but only 184 completed it until the end.^4^ We excluded participants who reported that they had been distracted during the task, that we should not use their data or who had already participated in a similar study. The final sample included 178 participants (*M*_age_ = 26.35, *SD* = 2.74).

### Results and Discussion

**FIGURE 2:**
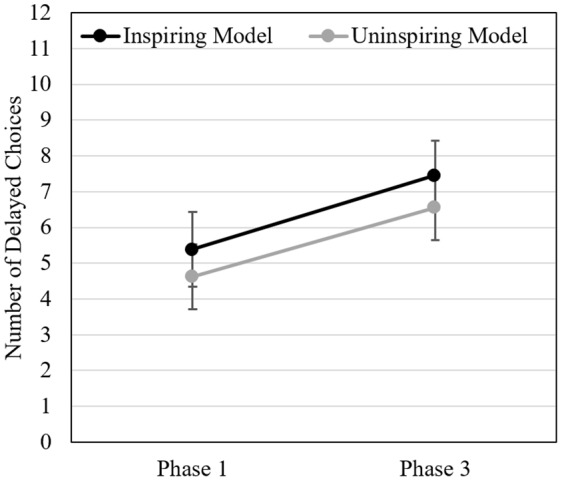
Study 2. Mean number of delayed choices before (Phase 1) and after exposure (Phase 3) to the inspiring vs. uninspiring model (*N* = 178). Error bars represent 95% between-subjects CIs.

We submitted the data to a 2 (within-subject: pre vs. post-exposure to the model) × 2 (between-subject: inspiring vs. uninspiring model) repeated measure ANOVA. Results are displayed in Figure 2. We found that participants opted for the delayed choice more often after than before having been exposed to the model [Wilk’s λ = 0.732, *F*(1, 176) = 64.358, *p* < 0.001, η*p*^2^ = 0.268, *d*_pooled_ = 0.604, *r*_repeated_ = 0.740]. However, contrary to our hypothesis, neither the main effect of the type of model [inspiring vs. uninspiring, *F*(1, 176) = 1.674, *p* = 0.197, η*p*^2^ = 0.009] nor the interaction between the two factors [Wilk’s λ > 0.999, *F*(1, 176) = 0.074, *p* = 0.786, η*p*^2^ < 0.001] revealed any significant effect. Moreover, participants did not report having thought more often about the inspiring model than the uninspiring model in Phase 3 on the modeling scale (*t*(176) = 1.51, *p* = 0.13, *d* = 0.227). Still, we found that the more participants reported having thought about the model in Phase 3 (i.e., higher modeling scale scores) the more they delayed in Phase 3 in comparison to Phase 1 (stronger difference between the number of delayed options chosen in Phase 3 compared to Phase 1; *r* (176) = 0.42, *p* < 0.001).

The absence of significant difference between the two modeling conditions did not seem, however, to be due to a failed manipulation of the perception participants had of the model. Manipulation checks indicated that, as expected, participants perceived the inspiring model as more likeable, similar, and competent (all *ts*(176) ≥ 3.768, *ps* < 0.001, *ds* ≥ 0.569) than the uninspiring model. Descriptive statistics and complementary analyses can be found in the Supplementary Material.

Results of this study suggest that simply observing an unknown person delay reward is enough to inspire one to do the same. These results may reflect a modeling influence. Still, they are difficult to interpret as the manipulation of the second factor, the description of the model, did not lead to any significant effect. It could thus be that the difference in ICs between pre and post-exposure to the model was not related to the model per se but to another parameter of the experiment, such as time (e.g., in Phase 3 participants could have reflected on the choices made in Phase 1 and change their behavior accordingly). We designed Study 3 to address this issue.

## Study 3

To confirm the hypothesis that the significant effects observed in Study 2 were related to the model, in Study 3, we manipulated the ICs made by the model.

### Methods

#### Procedure

The procedure of Study 3 was identical to the procedure of the inspiring model condition in Study 2 (see Figure 1) with the exception that in Phase 2 participants were exposed to either a *high delay model* who chose 10 times out of 12 the later larger reward or to *a low delay model* who chose only 2 times out of 12 the delayed option. Moreover, in addition to the likeability, similarity, competence and modeling scales used in Study 2, we asked participants in Phase 4 to which extent they tried to take the perspective of the model while they were watching her decisions in Phase 2 (simulation scale).

#### Participants

The study relied on a 2 (within-subject: pre vs. post-exposure to the model) × 2 (between-subject: high delay vs. low delay model) experimental design. We aimed for approximately 160 participants (80 per condition) to have the power to detect small effects of *r*∼0.11.

We collected the data of 283 female participants but 74 of them were incomplete. The inclusion criteria were the same as Study 2. We had to exclude 61 participants because they did not fulfill the gender (female participants only) or age (between 18 and 30 years old) inclusion criteria or because they indicated we should not use them. This left a final sample of 148 participants (*M*_age_ = 27.13, *SD* = 2.66). Participants received $1.50 settlement for completing the survey, which lasted about 15 min.

### Results and Discussion

**FIGURE 3:**
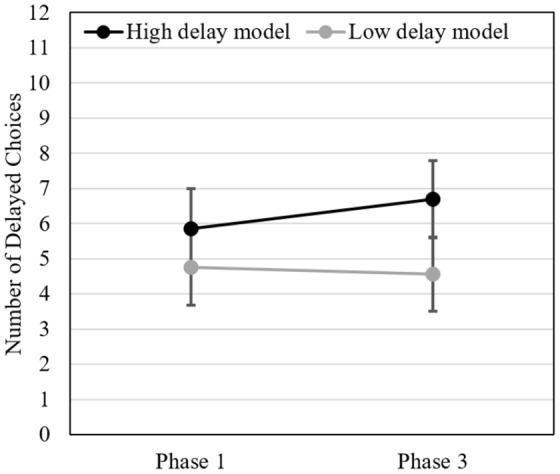
Study 3. Mean number of delayed choices before (Phase 1) and after exposure (Phase 3) to the high delay vs. low delay model (*N* = 148). Error bars represent 95% between-subjects CIs.

The 2 (between-subject: high delay vs. low delay model) × 2 (within-subject: pre vs. post-exposure to the model) repeated measure ANOVA revealed a main effect of the model’s IC style (*F*(1, 146) = 4.484, *p* = 0.036, η*p*^2^ = 0.032): participants delayed more in the high delay than in the low delay model condition. The main effect of exposure to the model (pre- vs. post-exposure) was not significant (Wilk’s λ = 0.979, *F*(1, 146) = 3.074, *p* = 0.082, η*p*^2^ = 0.021). But, as predicted, the interaction between the two factors was (Wilk’s λ = 0.948, *F*(1, 146) = 7.992, *p* = 0.005, η*p*^2^ = 0.052). Participants tended to delay more after having seen the high delay model and less after having seen the low delay model (see Figure 3). *Post hoc* comparisons indicated that this difference was significant in the high delay model condition (*t*(70) = 3.17, *p* = 0.002, *d*_pooled_ = 0.320, *r*_repeated_ = 0.853) but not in the low delay model condition (*t*(76) = 0.78, *p* = 0.440, *d*_pooled_ = 0.110, *r*_repeated_ = 0.932). *Post hoc* comparisons also indicated that although participants in the two experimental conditions did not significantly differ from each other in Phase 1 (*t*(146) = 1.39, *p* = 0.17, *d*_cohen_ = 0.23), participants exposed to a high delay model made more delayed choices in Phase 3 than participants exposed to a low delay model (*t*(146) = 2.75, *p* = 0.007, *d*_cohen_ = 0.45). Descriptive statistics and complementary analyses can be found in the Supplementary Material.

This study provides further evidence that modeling can exert a strong influence on people’s delay-of-reward behavior. We replicated the results of Study 2 showing that participants delayed more after having observed a high delay model. Moreover, we also found that this effect depended on the behavior of the model as revealed by the interaction between the two factors of our experimental design and the fact that participants who were exposed to the low delay model tended to delay less afterward. Interestingly, the difference in ICs between pre- and post-exposure to the low delay model did not reach significance. The data may thus suggest that modeling effects are stronger when the model is a high rather than a low delayer. However, participants could also have a tendency to delay more as time goes by and this effect of time may cancel the effects of modeling in the low delay model condition. To clarify this point, we would need a control condition devoid of any modeling influence, which is what we implemented in Study 4.

## Study 4

Study 4 had several objectives. The first one was to compare the modeling conditions created in the previous studies to control conditions that resembled them in every aspect except that there would not be any IC behavior to model. We thus added a factor to the design of Study 3 to create *relevant* vs. *irrelevant model* conditions.

The second objective of this study was to test whether the modeling effects created by this paradigm last over a longer period of time. In Bandura and Mischel’s (1965) experiment with children, modeling influences on ICs lasted up to 4 weeks after exposure to the model. In Study 4, we retested participants 3 months after the initial study and hypothesized that they would still exhibit modeling influences.

The third objective of this study was to investigate whether modeling influences carry over to another task likely to also involve delaying rewards. In Study 1, we found a correlation between ICs and procrastination tendencies. Thus, in Study 4, we put our participants in a situation in which they could be tempted to postpone performing a task and tested whether exposure to the model influenced the extent to which they procrastinated.

### Methods

We preregistered Study 4 on the Open Science Framework (see footnote 1).

#### Procedure

The procedure was composed of three parts corresponding to our three hypotheses (see Figure 1).

##### Part 1

The procedure of Part 1 followed the same procedure as Study 3 apart from a few elements. In the previous studies, we notably faced the issue that some participants were exposed to a model by which they could not be influenced because they already behaved in the pre-exposure IC task like her (e.g., high delay participants exposed to a high delay model). The data collected among these participants were thus irrelevant for our hypothesis.^5^ To avoid this problem, in Study 4, instead of randomly allocating participants to the model conditions, we decided to expose them to a model with an opposite IC style. We classified participants as high or low delayers based on their responses in Phase 1. For a clear cut-off point, we reduced the number of ICs from 12 to 11. Low delay participants, i.e., participants who chose 0 to 5 delayed options in Phase 1, were exposed in Phase 2 to a high delay model who chose 9 times out of 11 the delayed option. Conversely, high delay participants, i.e., participants who chose 6 to 11 delayed options in Phase 1, were exposed in Phase 2 to a low delay model who chose 2 times out of 11 the delayed option.

Moreover, we added an *irrelevant model* control condition in which participants had to observe decisions supposedly made by the model in a task that did not involve delaying reward: they were told that the model had to choose according to her preference between pairs of objects (e.g., a rain coat vs. an umbrella) and that they should memorize these decisions. The control conditions were similar to the experimental conditions regarding response localization. Indeed, in the *relevant model* conditions, the model mostly chose one side (e.g., the high delay model mostly picked the high delay option located on the right hand side). We created the irrelevant model conditions so that low delay participants would be exposed to a model choosing the object on the right side 9 times out of 11, and high delay participants would be exposed to a model choosing the object on the left side 9 times out of 11. Participants were randomly assigned to the relevant or irrelevant model conditions.

To sum up the procedure of Part 1, participants first made 11 ICs that enabled us to categorize them as high or low delay participants (Phase 1). Then, they had to observe and memorize the choices made by a relevant or irrelevant model (Phase 2). Next, they were asked again to make 11 ICs (Phase 3) and to recall the choices made by the model (Phase 4). Contrary to Studies 2 and 3, this study continued after Phase 4. Thus, to avoid raising participants’ suspicion regarding the goal of our research, we did not ask them in Phase 4 to rate the model and their thoughts about her but we invited them to participate in a Bonus Study to receive additional payment. This Bonus Study constituted the new task for which we expected to see carry over effects of the relevant model.

We told participants that this Bonus Study would only take a couple of minutes and would be generously compensated for the little effort it required. Participants were also told that they could only participate after having waited for at least 1 h. They then had up to 48 h to complete the study. We implemented this time constraint to put participants at risk of procrastinating. After reading the description of the Bonus Study, participants had to indicate with “yes” or “no” whether they intended to participate and generate an individual code for us to match their data and reward them accordingly on MTurk. Lastly, they typed in the time as it appeared on their computer and received a feedback indicating the time window during which they could complete the Bonus Study as well as a link to access it.

##### Part 2

Before arriving to the actual Bonus Study page, participants were notified that they should not proceed before 1 h had passed. If they indicated their wish to continue, they were then led to the Bonus Study. The click on this last link served as our dichotomous dependent measure as it indicated whether participants had been able to fulfill the task to which they had committed. The Bonus Study in itself consisted in three items assessing whether participants had procrastinated to complete this study. After which, they were thanked and offered a debriefing via email.

##### Part 3

Three months after Part 2, we invited participants to take part in an additional study, in which we asked them to complete another 11 ICs similar to those of Part 1. More detailed information concerning all the study material can be found in the Supplementary Material.

#### Participants

Power analyses were based on Part 2, which used a 2 (High vs. Low delay participants) × 2 (Relevant vs. Irrelevant model) design. This design includes two between-subject variables and thus required a larger sample than our other hypotheses, which also included within-subject variables. As common in social psychology studies, we expected the variables measured in Part 2 to display small to medium effect sizes. Hence, for an effect of a η*p*^2^ = 0.03 (*r*∼0.17), the power of 1−β = 0.8, α = 0.05, four groups, and numerator *df* = 1, approximately 260 participants were required. To account for a potential dropout rate between Part 1 and Part 2, we decided to aim for an initial sample of *N* = 400.

Our inclusion criteria were the same as in Studies 1 and 2: we aimed at recruiting female participants who had not previously participated in one of our studies, who indicated that they had not been distracted or only slightly distracted during the survey and that we should use their data. To make sure that we would be able to collect enough data to have sufficient power to test our hypotheses we extended the age range of our participants in comparison to Studies 2 and 3, i.e., from 18 to 40 years. In the end, 696 MTurk workers completed Part 1. Only 399 participants finished it and 29 of them had to be excluded because they did not fulfill our inclusion criteria, leaving a final sample of 370 participants (*M*_age_ = 30.57, *SD* = 4.87).

Participants received $1.50 settlement for their participation in the first part of our study, which lasted about 15 min. They received another $1 if they completed the Bonus Study according to the requirements. The time stamps recorded by the online survey platform Questback Unipark and participants’ reports indicated if they respected the time constraints (i.e., to complete the Bonus Study between 1 and 49 h after they had finished the first part).

### Results and Discussion

#### Part 1

We submitted the data to a 2 (between: low delay participants exposed to a high delay model vs. high delay participants exposed to a low delay model) × 2 (between: relevant vs. irrelevant model) × 2 (within: pre- vs. post-exposure to the model) repeated measure ANOVA. Results indicated that the three-way interaction we had hypothesized was significant (Wilk’s λ = 0.90, *F*(1, 366) = 40.05, *p* < 0.001, η*p*^2^ = 0.099). Inspection of the simple effects revealed that in the relevant model conditions, high delayers delayed less after exposure to a low delay model and low delayers delayed more after exposure to a high delay model (all *t*s ≥ 5.34, all *p*s < 0.001). In the irrelevant model conditions, none of this contrast reached significance (all *t*s ≤ 1.57, all *p*s ≥ 0.117). These results are thus in line with our previous studies and provide decisive evidence of the effects of modeling on delay-of-reward behavior.

#### Part 2

Three hundred thirty five participants from the initial sample indicated their willingness to complete the Bonus Study and 240 returned on time. This number is slightly lower than what we aimed for. Sensitivity analysis revealed that our sample was nevertheless big enough to detect small effects of η*p*^2^ > 0.032.

We performed two analyses. The first one was a logistic regression with participants’ return rate as dichotomous dependent variable, and their IC style (high vs. low delay participants), the model relevance, and the interaction term between these two factors as independent variables. Results indicated that participants’ IC style was the only significant predictor: high delay participants were more likely to return to the Bonus Study than were low delay participants (Wald’s χ^2^(1) = 7.09, *p* = 0.008, *OR* = 8.02). This suggests that the Bonus Study, as we had assumed, requires cognitive capacities that are also involved in ICs. However, neither the model relevance (Wald’s χ^2^(1) = 0.039, *p* = 0.843, *OR* = 0.96) nor the interaction term (Wald’s χ^2^(1) = 0.001, *p* = 0.975, *OR* = 1.02) significantly contributed to the explained variance. Hence, it seemed that the model did not influence participation to the Bonus Study.

Next, we investigated the answers to the procrastination scale that participants had completed in the Bonus Study with a 2 (participants’ IC style) × 2 (Model relevance) ANOVA. Neither the main effects nor the interaction led to any significant result [Intertemporal Choice Style: *F*(1, 236) = 0.008, *p* = 0.929, η*p*^2^ < 0.001; Model: *F*(1, 236) = 0.440, *p* = 0.508, η*p*^2^ = 0.002; Intertemporal Choice Style × Model: *F*(1, 236) = 0.605, *p* = 0.438, η*p*^2^ = 0.003]. Therefore, altogether these results did not provide evidence for carry over effects of modeling to a related but different task.

#### Part 3

**FIGURE 4:**
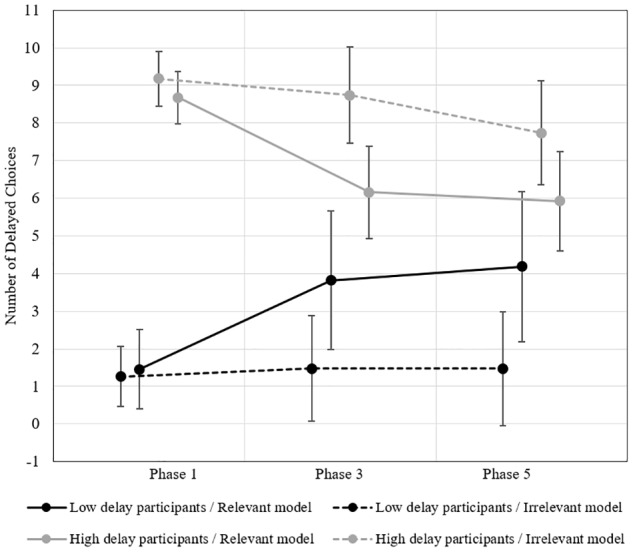
Study 4. Mean number of delayed choices before and after exposure to the high delay vs. low delay models (*N* = 78). Error bars represent 95% CIs.

78 participants of the initial sample responded to our request to perform again the IC task 3 months afterward, a number inferior to what we were aiming for. Sensitivity analysis revealed that this sample would be sufficient to detect a large effect (*r* = 0.31 or η*p*^2^ = 0.096, based on the smallest cell occupation of *n* = 11 and 80% power). Again, we submitted the data to a 2 (between-subject: low delay vs. high delay participants) × 2 (between-subject: relevant vs. irrelevant model) × 2 (within-subject: pre-exposure vs. 3 months after exposure) repeated measure ANOVA. Results revealed the significant three-way interaction we had hypothesized (Wilk’s λ = 0.94, *F*(1, 74) = 4.79, *p* = 0.032, η*p*^2^ = 0.061, see Figure 4). A similar pattern as in Part 1 emerged in the simple *post hoc* effects comparing the decisions at Phase 1 and Phase 5. In the relevant model conditions, high delayers delayed less 3 months after exposure to the low delay model than they did before having been exposed to her (*t*(24) = 3.74, *p* < 0.001, *d*_pooled_ = 0.352, *r*_repeated_ = 0.309). Conversely, low delayers in the relevant model condition delayed more 3 months after exposure to the high delay model than they did before exposure (*t*(10) = 2.45, *p* = 0.012, *d*_pooled_ = 0.119, *r*_repeated_ = 0.212). In the irrelevant model conditions, none of the contrasts between Phase 1 and Phase 5 reached significance (all *t*s < 1.87, all *p*s > 0.066). These results suggest a remarkable persistence of the modeling effects through time. However, we advise caution when interpreting this data given that the sample size was small and the variances grew larger over time.

## General Discussion

The present article reports a series of studies that build onto each other to show that being exposed to a person’s delay-of-reward behavior influences one’s own choices. Previous studies performed in the 1960s and 1970s have reported this effect in children and teenagers (Bandura and Mischel, 1965; Staub, 1972; Stumphauzer, 1972), two populations prone to identify with role models (Law and Hall, 2009). More recent research in adults replicated these results (Nicolle et al., 2012; Gilman et al., 2014; Calluso et al., 2017). Some of these studies even demonstrated that modeling exposure could influence participants’ delay discounting function (Garvert et al., 2015; Calluso et al., 2017; Devaine and Daunizeau, 2017). However, the setting required to implement these analyses made these paradigms remote from everyday life observational learning situations. Indeed, to gain sufficient statistical power, they relied on a high number of trials. Moreover, the goal to investigate social influence was obvious. In the studies reported in the present article, we thus decided to adopt a more simple and natural paradigm.

We created an online paradigm introduced to participants as an investigation into decision-making and memory. Participants were MTurk workers who did not have any direct contact with the model or the experimenters. They were just exposed for a few trials to the model’s choices and asked to memorize them. Moreover, we explicitly told them that they were no right or wrong decision and that they should decide on their own. However, although our modeling exposure was minimal, in all studies we found a consistent effect of modeling on ICs.

In Study 2, in which the model showed a preference for the delayed options (i.e., high delay model), we found that participants chose the delayed reward more often after than before having observed her decisions. In Study 3, we found that the direction of the influence depended on the model’s behavior: participants delayed more after having seen a high delay than a low delay model. Interestingly, the contrast between pre- and post-test was significant for participants exposed to a high delay model but it was not for participants exposed to a low delay model. However, this contrast became significant in Study 4, in which participants were exposed to model with a more different IC style. These result are in line with previous studies in children and adults (Bandura and Mischel, 1965; Nicolle et al., 2012; Gilman et al., 2014; Garvert et al., 2015; Moutoussis et al., 2016; Calluso et al., 2017; Devaine and Daunizeau, 2017). They suggest that the influence of a model can increase as well as decrease delay-of-reward behaviors, and thus that modeling can have both positive and negative consequences depending on the model’s behavior. Study 4 finally confirmed the effect of modeling on ICs in comparison to a control condition and showed that it was still significant 3 months after exposure to the model. These effects and their persistence in time point to a pervasive influence of modeling on delay-of-reward behavior.

Our studies present several limitations. Firstly, we did not find any generalization effect. In Study 4, we investigated whether modeling influences carried over to another task likely to also involve delaying rewards. To test this hypothesis, we put our participants in a situation in which they could be tempted to procrastinate and tested whether exposure to the model influenced the extent to which they performed the task to which they were committed. Procrastination involves delaying reward to the extent that immediate rewards (e.g., watching TV) receive higher preference than long-term goals (e.g., performing the Bonus Study in order to receive a supplementary reward). The Bonus study, seemed to be a valid measure for this purpose. Overall, high delay participants returned to the Bonus Study more consistently than low delay participants did. Still, we did not observe any influence of the model exposure.

Several characteristics of our paradigm may explain this absence of generalization effect. Firstly, our experimental manipulation may have been too subtle to engender changes deep enough to be extended to new situations. Modeling effects are known to be stronger for behaviors that are more similar (Bandura, 1986). Thus, our experimental manipulation may have suffice to create a near transfer (i.e., a modeling effect on a similar task) but it may have lacked power to generate far-transfers (i.e., a modeling effect on another task) especially on a task as different as the Bonus Study was from the IC. Another possibility is that our Bonus Study was not sensitive enough to capture a subtle effect. We used a single measure of procrastination (i.e., participants’ return to the Bonus study), which may have lacked reliability. Our paradigm thus needs to be developed and adapted to potentiate modeling effects on delay-of-reward and further explore the hypothesis of a generalization effect.

A second limitation of our studies relates to the facts that we only investigated female participants. We chose to focus on women because they are supposed to be more sensitive to social influences (Cacioppo and Petty, 1980). We decided to adopt this approach motivated by the desire to advance in small but reliable steps, building up knowledge one brick at a time. Our modeling samples were only composed of women but, on other respects, they were more diverse than usual psychology studies based on student samples: our age range was broader and our participants’ occupations, education and living areas more diversified. Future research should nevertheless confirm that modeling effects replicate in a male sample.

Thirdly, it is important to mention that we used hypothetical rewards. Our decision to rely on hypothetical rewards was based on previous studies that show that they are similarly discounted as real rewards (Madden et al., 2003; Locey et al., 2011). In line with this notion, we found that our hypothetical IC paradigm displayed significant correlations with several important everyday life indicators, such as education level, SES, BMI and smoking behavior. On could thus assume that modeling effects will replicate with actual money. Still this should be demonstrated.

A final limitation of our studies relates to the fact that to improve the ecological validity of our paradigm, we relied on a short IC task. Therefore, we did not have enough trials to calculate the delay discounting function underlying participants’ choices. Our results have thus to be interpreted with caution. We can argue that our participants’ ICs were influenced by those of the model. However, we cannot draw any conclusion regarding the mental model on which participants relied to make these choices. In other words, we cannot tell whether their discounting function changed after having observed the model. Previous studies using a higher number of trials have found that the social context can influence people’s discounting functions. People seem to adjust their discount rate when they observe or think about someone with a different discounting style (Garvert et al., 2015; Calluso et al., 2017; Devaine and Daunizeau, 2017) as well as when they are aware that someone observes their decisions (O’Brien et al., 2011; Weigard et al., 2014). The presence and the behavior of others has thus the capacity to deeply influence how people think about ICs. The current series of studies can only inform at the behavior level. Future research should investigate deeper processes.

The studies that we report in this article lay the ground for an approach to self-control improvement based on social influence. Psychological research has found that the ability to resist temptations and delay rewards is one of the most useful one. The first study that we reported in this article replicated this effect. The more people delay rewards the more likely they are to have a high education, a successful career and a healthy lifestyle. Relying on the power of inspiration that role models arouse could thus help people regain control over their lives.

## Ethics Statement

This study was carried out in accordance with the recommendations of the American Psychological Association. All subjects gave written informed consent in accordance with the Declaration of Helsinki. The protocol was approved by the Ethics Committee from the University of Graz.

## Author Contributions

All authors conceived the project hypotheses, designed the experimental material, and contributed to the writing of the manuscript. GK and HB collected the data and conducted the analyses.

## Conflict of Interest Statement

The authors declare that the research was conducted in the absence of any commercial or financial relationships that could be construed as a potential conflict of interest.

## Footnotes

1 https://osf.io/uu7cm/

2 These results correspond to two-tailed correlations uncorrected for multiple comparisons. The results remain the same if we apply a false discovery rate correction. The Benjamin-Hochberg adjusted *P* values for the correlations between the number of delayed choices on the one hand and on the other hand the procrastination score, the household income, the subjective SES and the number of cigarettes smoked per week are then all below or equal to 0.05 (0.05, 0.005, 0.05, and 0.05, respectively). The correlation between the number of delayed choices and the social anxiety score remains non-significant (*p* = 0.88).

3 To reinforce the belief in a real experimental partner, we asked participants at the very beginning of the study to enter the same information as the information provided by the model.

4 The number of participants who did not finish the survey may surprise the reader: it is high and higher than in Study 1. The reason for that is that we had stricter inclusion criteria (based on gender and age). We mentioned these criteria in our advertisement on MTurk. Still many people clicked on the online survey and started it although they were not eligible. After they had entered their demographical information, they were then automatically excluded from the survey before having even started Phase 1. Their data were thus incomplete and useless but they still figure in the raw data table, i.e., among the 823 initial participants. Raw and filtered data tables can be found on https://osf.io/uu7cm/.

5 Note that the analyses of Studies 2 and 3 reported above include all participants irrespectively of their IC style in Phase 1. In the Supplementary Material, we also report results of analyses performed in subsamples of participants who, in Phase 1, opted for less delayed choices than the model in the high delay model condition and more delayed choices than the model in the low delay model condition. These complementary analyses reveal similar results as those described in the main article.

## Abbreviations

BMI: body mass index
IC: intertemporal choice
MTurk: amazon’s mechanical turk
SIAS: social interaction anxiety scale
SES: socioeconomic status.
