# Supplementary material for: Improving Self-Control: The Influence of Role Models on Intertemporal Choices
Source: Front Psychol. 2019 Aug 2;10:1722. doi: 10.3389/fpsyg.2019.01722 (PMC6688538; doi:10.3389/fpsyg.2019.01722)
Supplement: Supplementary file 1 [file Table_1.docx]

**Supplementary Material and Results**

**Study 1**

**Methods**

**Material.**

***Intertemporal choice (IC) offers*.** The modeling paradigm we relied on (Studies 2-4) requires participants to perform several sessions of IC (i.e. before and after exposure to the model). Thus, we used this first study to pretest and validate two sets of offers that slightly differed from each other (see supplementary Table 1). Participants were randomly assigned to one or the other set. The offers within each set were presented in a random order.

Supplementary Table 1

*Study 1. Intertemporal choice offers.*

| Small set (N = 92) | Large set (N = 94) |
| --- | --- |
| $505 in 1 week vs $520 in 2 weeks | $525 in 1 week vs $540 in 2 weeks |
| $520 in 2 weeks vs $535 in 3 weeks | $540 in 2 weeks vs $555 in 3 weeks |
| $505 in 1 week vs $535 in 3 weeks | $525 in 1 week vs $555 in 3 weeks |
| $505 in 1 week vs $550 in 4 weeks | $525 in 1 week vs $570 in 4 weeks |
| $550 in 4 weeks vs $590 in 7 weeks | $570 in 4 weeks vs $615 in 7 weeks |
| $505 in 1 week vs $590 in 7 weeks | $525 in 1 week vs $615 in 7 weeks |
| $155 in 1 week vs $160 in 2 weeks | $175 in 1 week vs $180 in 2 weeks |
| $160 in 2 weeks vs $165 in 3 weeks | $180 in 2 weeks vs $185 in 3 weeks |
| $155 in 1 week vs $165 in 3 weeks | $175 in 1 week vs $185 in 3 weeks |
| $155 in 1 week vs $170 in 4 weeks | $175 in 1 week vs $190 in 4 weeks |
| $170 in 4 weeks vs $185 in 7 weeks | $190 in 4 weeks vs $205 in 7 weeks |
| $155 in 1 week vs $185 in 7 weeks | $175 in 1 week vs $205 in 7 weeks |

Before answering Phase 1 offers, participants had the possibility to get familiar with the task in two practice trials involving different offers than the trials of interest ($590 in 7 weeks vs $605 in 8 weeks; $540 in 2 weeks vs $570 in 4 weeks).

**Results**

**Comparison of the Two sets of Offers.** We did not find any significant difference between the number of delayed choices made by the participants who were assigned to the small set of offers (M = 5.43, SD = 4.66) compared to those who were assigned to the large set of offers (M = 5.53, SD = 4.98, t(184) = 0.137, p = .89, d = 0.02).

Supplementary Table 2

| Scales and continuous measurements | Mean (SD) |
| --- | --- |
| Pure Procrastination Scale | 2.24 (0.96) |
| Social Interaction Anxiety Scale | 1.44 (0.95) |
| Smoking behavior (results for smoker participants, N = 29)  Total months of smoking  Number of cigarettes per day on average  Smoking procrastination score | 185.03 (137.24)  14.17 (6.61)  3.64 (1.31) |
| Subjective Socioeconomic Status | 4.70 (1.80) |

*Study 1. Descriptive statistics for the scales and continuous measurements (N = 186).*

**Descriptive Statistics and Complementary Analyses.** We report the descriptive statistics for the scales and continuous measurements in Supplementary Table 2 and those for categorical variables in Supplementary Table 3.

Supplementary Table 3

*Study 1. Descriptive statistics for the ordinal and categorical variables (N = 186)*

| Categorical variables | N | Mean number of delayed choices (SD) |
| --- | --- | --- |
| Are you smoking?  Yes  Occasionally  No | 29  22  135 | 3.21 (3.71)  5.68 (4.98)  5.94 (4.89) |
| Weight status  Underweight (BMI < 18.5)  Healthy (18.5 ≤ BMI < 24.9)  Overweight (25 ≤ BMI < 29.9)  Obese (BMI > 30) | 4  81  52  48 | 3.00 (6.00)  5.98 (4.96)  6.04 (4.85)  4.38 (4.30) |
| Income  < $25,000  $25,000 ≤ income < $50,000  $50,000 ≤ income < $75,000  $75,000 ≤ income < $100,000  $100,000 ≤ income < $150,000  ≥ $150,000 | 35  51  46  29  19  6 | 4.46 (4.87)  4.12 (4.50)  5.98 (4.81)  6.48 (4.85)  7.16 (4.54)  9.17 (4.22) |
| Education level  School  High school  College  Bachelor  Master  PhD | 0  52  52  58  21  3 | -  4.06 (4.52)  5.12 (4.78)  6.31 (4.94)  7.62 (4.37)  5.67 (5.51) |

***Smoking behavior*.** Our sample was composed in majority of non-smokers (see Supplementary Table 3). In the ANOVA reported in the main article, we compared the number of delayed choices made by the participants belonging to the three categories of smoking behavior we assessed (smokers, occasional smokers, non-smokers). However, the number of participants in each cell was highly unequal. Therefore, we decided to compare the smokers to a subsample of non-smokers equal in size and matched for age, gender (12 women) and education level. Here again, results indicated that smokers tended to delay less than non-smokers (see Supplementary Table 4).

Supplementary Table 4

*Study 1. Comparison of smokers (N = 29) to their matched non-smokers (N = 29)*

|  | Mean smokers (SD) | Mean non-smokers (SD) | Statistics |
| --- | --- | --- | --- |
| Age | 36.59 (10.49) | 36.90 (9.94) | t(56) = 0.12, *p* = .91, d = 0.03 |
| Education level | 3.03 (1.21) | 3.00 (1.13) | t(56) = 0.11, *p* = .91, d = 0.03 |
| N delayed choices | 3.21 (3.71) | 6.41 (4.93) | t(56) = 2.80, *p* = .007, d = 0.73 |

***Objective Socioeconomic Status*.** The results reported in the main article depict the correlation between the number of chosen delayed options and participants’ income. However, participants were asked to report the income of their family household. Therefore, we decided to perform an additional OLS regression analysis regressing the number of delayed choices on the income variable, while controlling for the number of people in the household. The effect of income on the number of delayed choices remained significant (*b* = -0.324, *t*(183) = -1.515, *p* < .001, *r_partial_* = -.111).

**Study 2**

**Methods**

**Material**

***Intertemporal choice offers*.** For Phase 1 and Phase 3 (i.e. when participants were asked to make intertemporal choices), we used the same two sets of offers as in Study 1. Half of the participants were randomly assigned to the small set in Phase 1 and the large set in Phase 3, and the other half were assigned to the opposite order (i.e. large set in Phase 1 and small set in Phase 3). In addition, we used for Phase 2 (i.e. the decisions made by the model) another set of offers involving rewards which values were in between the small and large sets of offers (see Supplementary Table 5). The offers within each set were presented in a random order.

Supplementary Table 5

*Study 2. Intertemporal choice offers.*

| Small set  (choices made by participants in either Phase 1 or Phase 3) | Medium set  (choices made by the model) | Large set  (choices made by participants in either Phase 1 or Phase 3) |
| --- | --- | --- |
| $505 in 1 week vs $520 in 2 weeks | $515 in 1 week vs **$530 in 2 weeks** | $525 in 1 week vs $540 in 2 weeks |
| $520 in 2 weeks vs $535 in 3 weeks | $530 in 2 weeks vs **$545 in 3 weeks** | $540 in 2 weeks vs $555 in 3 weeks |
| $505 in 1 week vs $535 in 3 weeks | $515 in 1 week vs **$545 in 3 weeks** | $525 in 1 week vs $555 in 3 weeks |
| $505 in 1 week vs $550 in 4 weeks | **$515 in 1 week** vs $560 in 4 weeks | $525 in 1 week vs $570 in 4 weeks |
| $550 in 4 weeks vs $590 in 7 weeks | $560 in 4 weeks vs **$605 in 7 weeks** | $570 in 4 weeks vs $615 in 7 weeks |
| $505 in 1 week vs $590 in 7 weeks | $515 in 1 week vs **$605 in 7 weeks** | $525 in 1 week vs $615 in 7 weeks |
| $155 in 1 week vs $160 in 2 weeks | $165 in 1 week vs **$170 in 2 weeks** | $175 in 1 week vs $180 in 2 weeks |
| $160 in 2 weeks vs $165 in 3 weeks | $170 in 2 weeks vs **$175 in 3 weeks** | $180 in 2 weeks vs $185 in 3 weeks |
| $155 in 1 week vs $165 in 3 weeks | $165 in 1 week vs **$175 in 3 weeks** | $175 in 1 week vs $185 in 3 weeks |
| $155 in 1 week vs $170 in 4 weeks | $165 in 1 week vs **$180 in 4 weeks** | $175 in 1 week vs $190 in 4 weeks |
| $170 in 4 weeks vs $185 in 7 weeks | $180 in 4 weeks vs **$195 in 7 weeks** | $190 in 4 weeks vs $205 in 7 weeks |
| $155 in 1 week vs $185 in 7 weeks | **$165 in 1 week** vs $195 in 7 weeks | $175 in 1 week vs $205 in 7 weeks |

Note: The options selected by the model are marked in bold font.

***Experimental manipulation*.** Phase 2, i.e. the modeling phase, was presented as a memory test. We told participants that they would be matched with an experimental partner who already had completed the study. They would see her answers to a similar task than the one they had just performed and would have to pay attention to her information as well as the intertemporal choices she had made as we would later test their memory. Participants were then shown 12 female names. In the inspiring model condition, we asked them to choose the name of the person they would like to have as experimental partner. We chose these names to be plausible, i.e. we mostly chose names frequent in the generation to which the model was supposedly belonging (i.e. American women in their 20s or 30s). We also added a few rarer names so that it would not look too constructed. We assumed that participants would select a name that they liked and that this would foster modeling effects. In the uninspiring model condition, we told them that the computer would randomly choose the name of the person that would be their experimental partner.

Then, they were presented with a short description of the experimental partner including her name (chosen by the participant in the inspiring model condition vs a participant called “Rada” in the uninspiring model condition), age (one year older than the participant in the inspiring model condition vs one year younger in the uninspiring model condition), gender (female in both conditions), nationality (American in the inspiring model condition vs Russian in the uninspiring model condition), location (USA in both conditions), English as mother tongue (“yes*”* in the inspiring model condition vs “no” in the uninspiring model condition), educational level (same educational level as the participant in the inspiring model condition vs high-school level in the uninspiring model condition). Below this description, participants could read a message written by their experimental partner for them. The message in the inspiring model condition was:

*“I wish you good luck with the task! I enjoyed it a lot. A shame that it was not real money! Usually I am quite good at making financial decisions. I must admit that it wasn’t always easy to choose between the different options, but in general, I am the kind of person who rather waits for a later better benefit. In my opinion, it’s worth waiting a bit to get more money in the end.”*

The message in the uninspiring model condition was:

*“Im supposed to write something but I don’t know you and I don’t have much to say. I cant give you any advice about the task, I usually suck at making money decisions! Pff… what a pain in the a** to chose between the different options! In general Im the kind of person whod go for the bigger benefit. I think waiting a bit for money is ok if you get more out of it.”*

The messages were followed by the presentation of the decisions made by the model. The screens look identical to the screens presented to the participants in Phases 1 and 3, except that this time one of the two options was automatically selected, supposedly reflecting the decision made by the experimental partner.

***Manipulation checks and modeling scale***. At the end of the study (i.e. Phase 4), participants were asked to recall three of the decisions made by the model and to assess 1) her similarity (“My experimental partner and I seem to be quite similar”, “My experimental partner and I seem to have a lot in common”, “I could imagine that my experimental partner and I have similar personalities”; Cronbach’s α = .95), 2) her competence with money (“My experimental partner seems to be able to make reflected decisions when it comes to money”, “My experimental partner seems to be competent with money”, “My experimental partner seems to be good at making long term money decisions”; Cronbach’s α = .90), and 3) her likeability (“My experimental partner seems to be a nice person”, “I have a positive impression of my experimental partner”, My experimental partner seems to be quite likeable”; Cronbach’s α = .96). In addition, we asked participants to answer 3 modeling items (“Watching the decisions made by my experimental partner made me think differently about my own decisions”, “In the third run of the study, I’ve thought several times about what my experimental partner would have done”, “In the third run of the study, I’ve drawn inspiration from my experimental partner for my own decisions”; Cronbach’s α = .89). All items were assessed on 7-point scale (1: strongly disagree, 7: strongly agree).

**Results**

**Comparison of the Two Sets of Offers.** We did not find any significant difference between the number of delayed choices made by the participants who were assigned to the small or large sets of offers, neither in Phase 1 (M_Small_ = 4.81, SD_Small_ = 4.52, M_Large_ = 5.11, SD_Large_ = 4.79, t(176) = 0.42, p = .67, d = 0.064) nor in Phase 3 (M_Small_ = 6.96, SD_Small_ = 4.71, M_Large_ = 6.98, SD_Large_ = 4.35, t(176) = 0.03, p = .98, d = 0.004).

**Descriptive Statistics and Complementary Analyses.** We report the descriptive statistics in Supplementary Table 6.

Supplementary Table 6

*Study 2. Means (SD) of the manipulation checks and modeling scales in the two experimental conditions.*

| Scales | Inspiring model  (N = 81) | Uninspiring model (N = 97) | Statistics |
| --- | --- | --- | --- |
| Similarity | 4.58 (1.58) | 3.69 (1.55) | *t*(176) = 3.77, *p* < .001, *d* = 0.57 |
| Competence | 5.56 (1.22) | 4.72 (1.40) | *t*(176) = 4.23, *p* < .001, *d* = 0.64 |
| Likeability | 5.85 (1.01) | 5.03 (1.34) | *t*(176) = 4.51, *p* < .001, *d* = 0.68 |
| Modeling | 3.65 (1.64) | 3.30 (1.53) | *t*(176) = 1.51, *p* = .13, *d* = 0.22 |


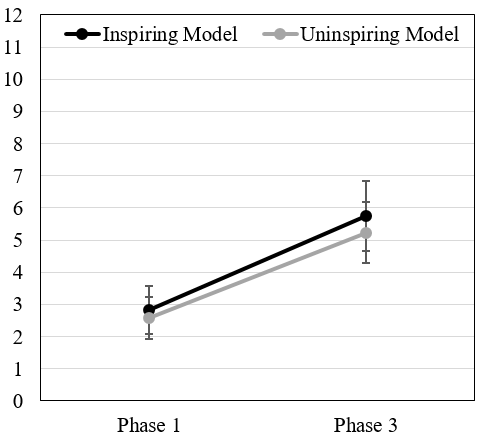
***2 x 2 ANOVA***. Only the participants who were low delayers in Phase 1 could change their delay-of-reward behavior in the direction of the high delay model. Therefore, we decided to rerun the 2 (pre vs post-exposure to the model) x 2 (inspiring vs uninspiring model) ANOVA with the participants who had chosen 9 or less delayed options in Phase 1 and could thus adjust their behaviors to the model who had chosen the delayed option 10 times out of 12. This supplementary analysis revealed the same pattern of results as the ANOVA reported in the main article (see Supplementary Figure 1). The effect of exposure to the model remained significant (Wilk’s λ = .598, *F*(1, 131) = 87.967, *p* < .001, η*p*² = .402) but neither the type of model (inspiring vs uninspiring : *F*(1, 131) = 0.483, *p* = .488, η*p*² = .004) nor the interaction between the two experimental factors (Wilk’s λ = .999, *F*(1, 131) = 0.193, *p* = .662, η*p*² = .001) led to any significant results.

Supplementary Figure 1. Study 2. Mean delayed choices before (Phase 1) and after exposure (Phase 3) within the selected sample of participants (N = 133). Error bars represent 95% between-subjects CIs.

Moreover, one could argue that participants who had a low education level were more inspired by the uninspiring model who also had a low education level and that this could explain why we did not find any difference between the two experimental conditions. To rule out this explanation, we have rerun the 2 (model: inspiring vs uninspiring) x 2 (pre- vs post-exposure) ANOVA in the subsample of participants whose education level was higher than high school, i.e. the model’s education level (N = 140). The results are similar to those observed in the big sample, i.e. a main effect of exposure (F(1, 138) = 46.83, p < .001, ηp² = .25) but no significant effect of the type of model or interaction (all Fs < 0.33, p > .57, ηp² < .001). It thus seems unlikely that this factor accounts for the non-significant difference between the two types of models.

***Correlations***. Correlations between the change in the number of delayed choices between Phase 1 and Phase 3 and the scales assessed in Phase 4 only revealed significant results for the modeling items (see Supplementary Table 7 and Supplementary Figure 2).

Supplementary Table 7

*Study 2. Correlations coefficients*

|  | Similarity | Competence | Likeability | Modeling |
| --- | --- | --- | --- | --- |
| Change in delayed choices  (N_Phase 3_ – N_Phase 1_) | .02 | -.02 | .07 | .42** |
| Similarity |  | .55** | .35** | .19** |
| Competence |  |  | .50** | .17* |
| Likeability |  |  |  | .18* |

Note: * *p* < .05; ** *p* < .01


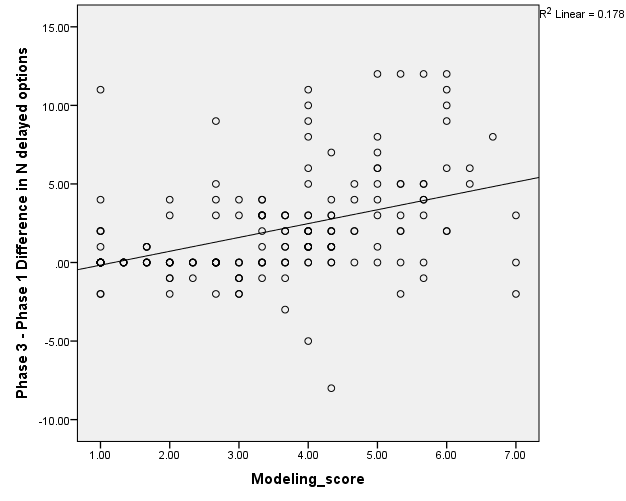


Supplementary Figure 2. Study 2. Scatter plot describing the change in delayed choices as a function of the modeling score.

**Study 3**

**Methods**

**Material**

***Intertemporal choice offers*.** We used the same offers as in Study 2. The offers received by the participants are displayed in Supplementary Table 8.

Supplementary Table 8

*Study 3. Intertemporal choice offers in Phases 1 & 3 (decisions made by the participants)*

| Small set | Large set |
| --- | --- |
| $505 in 1 week vs $520 in 2 weeks | $525 in 1 week vs $540 in 2 weeks |
| $520 in 2 weeks vs $535 in 3 weeks | $540 in 2 weeks vs $555 in 3 weeks |
| $505 in 1 week vs $535 in 3 weeks | $525 in 1 week vs $555 in 3 weeks |
| $505 in 1 week vs $550 in 4 weeks | $525 in 1 week vs $570 in 4 weeks |
| $550 in 4 weeks vs $590 in 7 weeks | $570 in 4 weeks vs $615 in 7 weeks |
| $505 in 1 week vs $590 in 7 weeks | $525 in 1 week vs $615 in 7 weeks |
| $155 in 1 week vs $160 in 2 weeks | $175 in 1 week vs $180 in 2 weeks |
| $160 in 2 weeks vs $165 in 3 weeks | $180 in 2 weeks vs $185 in 3 weeks |
| $155 in 1 week vs $165 in 3 weeks | $175 in 1 week vs $185 in 3 weeks |
| $155 in 1 week vs $170 in 4 weeks | $175 in 1 week vs $190 in 4 weeks |
| $170 in 4 weeks vs $185 in 7 weeks | $190 in 4 weeks vs $205 in 7 weeks |
| $155 in 1 week vs $185 in 7 weeks | $175 in 1 week vs $205 in 7 weeks |

The choices made by the model are reported in Supplementary Table 9.

Supplementary Table 9

*Study 3. Phase 2 offers (decisions made by the model)*

| High delay model | Low delay model |
| --- | --- |
| $515 in 1 week vs **$530 in 2 weeks** | **$515 in 1 week** vs $530 in 2 weeks |
| $530 in 2 weeks vs **$545 in 3 weeks** | **$530 in 2 weeks** vs $545 in 3 weeks |
| $515 in 1 week vs **$545 in 3 weeks** | $515 in 1 week vs **$545 in 3 weeks** |
| **$515 in 1 week** vs $560 in 4 weeks | **$515 in 1 week** vs $560 in 4 weeks |
| $560 in 4 weeks vs **$605 in 7 weeks** | $560 in 4 weeks vs **$605 in 7 weeks** |
| $515 in 1 week vs **$605 in 7 weeks** | **$515 in 1 week** vs $605 in 7 weeks |
| $165 in 1 week vs **$170 in 2 weeks** | **$165 in 1 week** vs $170 in 2 weeks |
| $170 in 2 weeks vs **$175 in 3 weeks** | **$170 in 2 weeks** vs $175 in 3 weeks |
| $165 in 1 week vs **$175 in 3 weeks** | **$165 in 1 week** vs $175 in 3 weeks |
| $165 in 1 week vs **$180 in 4 weeks** | **$165 in 1 week** vs $180 in 4 weeks |
| $180 in 4 weeks vs **$195 in 7 weeks** | **$180 in 4 weeks** vs $195 in 7 weeks |
| **$165 in 1 week** vs $195 in 7 weeks | **$165 in 1 week** vs $195 in 7 weeks |

Note: The options selected by the models are marked in bold font.

***Simulation scale*.** Previous research suggests that people learn from others by simulating their actions and cognitive processes (Grafton, 2009). Thus, we assessed the extent to which participants tried to take the perspective of the model while watching her decisions. To that purpose we created 4 items (“While watching my experimental partner…” “… I tried to put myself in her shoes”, “… I tried to look at the task the way she did”, “… I tried taking her perspective”, “… I was wondering what motivated her to make these decisions”; Cronbach’s α = .91). Items were assessed on 7-point scale (1: strongly disagree, 7: strongly agree).This simulation scale was presented at Phase 4 along with the similarity (Cronbach’s α = .95), competence (Cronbach’s α = .91), likeability (Cronbach’s α = .93) and modeling scales (Cronbach’s α = .86) described in Study 2^[[1]](#footnote-1)^.

***Experimental manipulation*.** The instructions given to the participants were the same as in Study 2. Moreover, the model was introduced in the same way as in the inspiring condition of Study 2. For both the high and low delay models, participants could choose the name of their experimental partner among a list. Then, they were presented with a short description including her name, her age (one year older than the participant), gender (female), nationality (American), location (USA), English as mother tongue (“yes*”*), educational level (same educational level as the participant). Below this description, participants could read a message from their experimental partner for them.

The message in the high delay model condition was the same as in Study 2:

*“I wish you good luck with the task! I enjoyed it a lot. A shame that it was not real money! Usually I am quite good at making financial decisions. I must admit that it wasn’t always easy to choose between the different options, but in general, I am the kind of person who rather waits for a later better benefit. In my opinion, it’s worth waiting a bit to get more money in the end.”*

The message in the low delay model condition was similar as for the high delay model condition, except that the model here justified her interest for smaller sooner rewards:

*“I wish you good luck with the task! I enjoyed it a lot. A shame that it was not real money! Usually I am quite good at making financial decisions. I must admit that it wasn’t always easy to choose between the different options, but in general, I am the kind of person who prefers receiving a slightly smaller amount in shorter time. In my opinion, it’s not worth waiting to get a bit more money in the end.”*

The messages were followed by the presentation of the decisions made by the model. The screens look identical to the screens presented to the participants in Phases 1 and 3, except that this time one of the two options was automatically selected, supposedly reflecting the decision made by the experimental partner.

**Results**

**Comparison of the Two Sets of Offers.** We did not find any significant difference between the number of delayed choices made by the participants who were assigned to the small or large sets of offers, neither in Phase 1 (M_Small_ = 5.09, SD_Small_ = 4.51, M_Large_ = 5.49, SD_Large_ = 5.24, t(138.70) = 0.50, p = .62, d = 0.082) nor in Phase 3 (M_Small_ = 5.49, SD_Small_ = 5.24, M_Large_ = 5.66, SD_Large_ = 4.42, t(137.44) = 0.21, p = .83, d = 0.035).

**Descriptive Statistics and Complementary Analyses.** We report Study 3 descriptive statistics in Supplementary Table 10.

Supplementary Table 10

*Study 3. Means (SD) of the manipulation checks, modeling and simulation scales in the two experimental conditions.*

| Scales | High delay model  (N = 71) | Low delay model (N = 77) | Statistics |
| --- | --- | --- | --- |
| Similarity | 4.36 (1.80) | 4.74 (1.64) | *t*(146) = 1.35, *p* = .18, *d* = 0.22 |
| Competence | 5.37 (1.35) | 4.76 (1.57) | *t*(145.39) = 2.51, *p* = .01, *d* = 0.42 |
| Likeability | 5.92 (1.00) | 5.62 (0.96) | *t*(146) = 1.90, *p* = .06, *d* = 0.31 |
| Modeling | 3.39 (1.71) | 3.28 (1.70) | *t*(146) = 0.40, *p* = .69, *d* = 0,07 |
| Simulation | 4.62 (1.49) | 4.56 (1.63) | *t*(146) = 0.27, *p* = .79, *d* = 0.04 |

***2 x 2 ANOVA within the sample of participants who could have been influenced by the model***. As in the previous study, in Study 3 only participants who were different from the model in Phase 1 could display signs of influence in Phase 3 (i.e. delay more for those exposed to a high delay model and less for those exposed to a low delay model). Therefore, in this study again we decided to rerun the 2 (pre vs post-exposure to the model) x 2 (high delay vs low delay model) ANOVA with the subsample of participants who could adjust their delay-of-reward behavior to the model’s (i.e. who had chosen 9 or less delayed options in Phase 1 for those in the high delay model condition, and 3 or more delayed options for those in the low delay model condition). This supplementary analysis revealed the same pattern of results as the ANOVA reported in the main article. The main effect of exposure to the model remained non-significant (Wilk’s λ = .977, *F*(1, 85) = 2.026, *p* = .158, η*p*² = .023) but both the main effect of the model’s delaying style (high vs low delay : *F*(1, 85) = 43.841, *p* < .001, η*p*² = .340) and the interaction between the two experimental factors (Wilk’s λ = .865, *F*(1, 85) = 13.30, *p* < .001, η*p*² = .135) were significant (see Supplementary Figure 3). Post-hoc comparisons indicated that the difference between pre and post-exposure to the model was significant in the high delay model condition (*t*(46) = 3.74, *p* < .001, *d_pooled_* = 0.503, *r_repeated_* = .682) but not in the low delay model condition (*t*(39) = 1.51, *p* = .132, *d_pooled_* = 0.287, *r_repeated_* = .823).


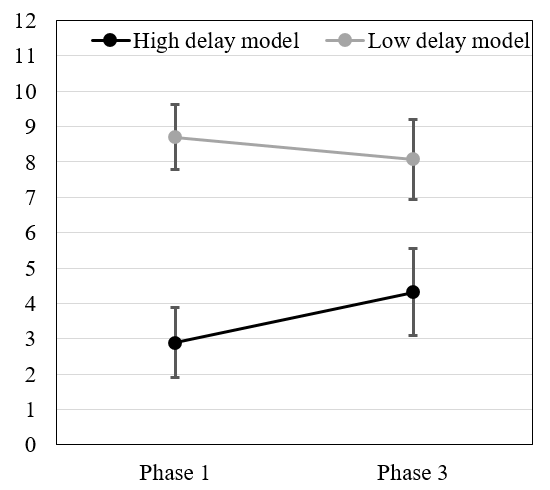


Supplementary Figure 3. Study 3. Mean delayed choices before (Phase 1) and after exposure (Phase 3) within the selected sample of participants (N = 87). Error bars represent 95% between-subjects CIs.

Moreover, as in Study 2, we found that the more participants reported having thought about the model in Phase 3 (i.e. higher modeling scale scores) the stronger the modeling influence (see Supplementary Tables 11 and 12 for all correlation results). Indeed, in the high delay condition, we found a positive correlation between the modeling score and the number of delayed options chosen in Phase 3 as compared to Phase 1 (r (45) = .29, *p* = .05). Conversely, in the low delay condition, we found a negative correlation between the modeling score and the number of delayed options chosen in Phase 3 as compared to Phase 1 (r (39) = -.31, *p* = .05).

Supplementary Table 11

*Study 3. Correlations coefficients in the high delay model condition within the selected sample of participants (N = 87)*

|  | Similarity | Competence | Modeling | Likeability | Simulation |
| --- | --- | --- | --- | --- | --- |
| Change in delayed choices  (N_Phase 3_ – N_Phase 1_) | .09 | -.14 | .29* | -15 | -.03 |
| Similarity |  | .64** | .34* | .33* | .32* |
| Modeling |  |  | .36* | .48** | .35* |
| Competence |  |  |  | .12 | .44** |
| Likeability |  |  |  |  | .26 |

Note: * *p* ≤ .05; ** *p* < .01

Supplementary Table 12

*Study 3. Correlations coefficients in the low delay model condition within the selected sample of participants (N = 87)*

|  | Similarity | Competence | Modeling | Likeability | Simulation |
| --- | --- | --- | --- | --- | --- |
| Change in delayed choices  (N_Phase 3_ – N_Phase 1_) | -.004 | -.003 | -.31* | .05 | -.16 |
| Similarity |  | .68** | .58** | .44** | .38* |
| Modeling |  |  | .50** | .38* | .33* |
| Competence |  |  |  | .37* | .48** |
| Likeability |  |  |  |  | .47** |

Note: * *p* ≤ .05; ** *p* < .01

**Study 4**

**Methods**

**Material**

***Intertemporal choice offers*.** Studies 1, 2 and 3 revealed no significant difference between the choices made in response to the small versus large sets of offers. Therefore, in Study 4, we let go of the two orders and decided to always present the small set offer in Phase 1 and the large set of offers in Phase 3. In addition, we removed from each set one trial to end up with a total number of 11 offers and split the range of possible answers into two equal parts (low delayers: 1 to 5 delayed choices, high delayers: 6 to 11 delayed choices; see Supplementary Table 13).

The offers presented in Phase 5 included for one-third offers from Phase 1, for another third offers from Phase 2 and for the last third offers from Phase 3. We assumed that at Phase 5 participants would not remember the offers from Phases 1, 2 and 3, particularly given that they then did not know that we would contact them again for another test three months later.

Supplementary Table 13

*Study 4. Intertemporal choice offers in Phases 1, 3 and 5 (decisions made by the participants)*

| Phase 1  (Small set) | Phase 3  (Large set) | Phase 5 |
| --- | --- | --- |
| $505 in 1 week vs $520 in 2 weeks | $525 in 1 week vs $540 in 2 weeks | $505 in 1 week vs $520 in 2 weeks |
| $520 in 2 weeks vs $535 in 3 weeks | $540 in 2 weeks vs $555 in 3 weeks | $540 in 2 weeks vs $555 in 3 weeks |
| $505 in 1 week vs $535 in 3 weeks | $525 in 1 week vs $555 in 3 weeks | $515 in 1 week vs $545 in 3 weeks |
| $505 in 1 week vs $550 in 4 weeks | $525 in 1 week vs $570 in 4 weeks | $505 in 1 week vs $550 in 4 weeks |
| $550 in 4 weeks vs $590 in 7 weeks | $570 in 4 weeks vs $615 in 7 weeks | $570 in 4 weeks vs $615 in 7 weeks |
| $505 in 1 week vs $590 in 7 weeks | $525 in 1 week vs $615 in 7 weeks | $515 in 1 week vs $605 in 7 weeks |
| $155 in 1 week vs $160 in 2 weeks | $175 in 1 week vs $180 in 2 weeks | $155 in 1 week vs $160 in 2 weeks |
| $160 in 2 weeks vs $165 in 3 weeks | $180 in 2 weeks vs $185 in 3 weeks | $180 in 2 weeks vs $185 in 3 weeks |
| $155 in 1 week vs $165 in 3 weeks | $175 in 1 week vs $185 in 3 weeks | $165 in 1 week vs $175 in 3 weeks |
| $155 in 1 week vs $170 in 4 weeks | $175 in 1 week vs $190 in 4 weeks | $155 in 1 week vs $170 in 4 weeks |
| $155 in 1 week vs $185 in 7 weeks | $175 in 1 week vs $205 in 7 weeks | $175 in 1 week vs $205 in 7 weeks |

The offers and choices made by the relevant model were the same as in Study 3, except for one that we removed to end up with 11 offers (see Supplementary Table 14).

Supplementary Table 14

*Study 4. Phase 2 offers in the experimental conditions: Decisions made by the relevant models*

| High delay model / Low delay participants | Low delay model / High delay participants |
| --- | --- |
| $515 in 1 week vs **$530 in 2 weeks** | **$515 in 1 week** vs $530 in 2 weeks |
| $530 in 2 weeks vs **$545 in 3 weeks** | **$530 in 2 weeks** vs $545 in 3 weeks |
| $515 in 1 week vs **$545 in 3 weeks** | $515 in 1 week vs **$545 in 3 weeks** |
| **$515 in 1 week** vs $560 in 4 weeks | **$515 in 1 week** vs $560 in 4 weeks |
| $560 in 4 weeks vs **$605 in 7 weeks** | $560 in 4 weeks vs **$605 in 7 weeks** |
| $515 in 1 week vs **$605 in 7 weeks** | **$515 in 1 week** vs $605 in 7 weeks |
| $165 in 1 week vs **$170 in 2 weeks** | **$165 in 1 week** vs $170 in 2 weeks |
| $170 in 2 weeks vs **$175 in 3 weeks** | **$170 in 2 weeks** vs $175 in 3 weeks |
| $165 in 1 week vs **$175 in 3 weeks** | **$165 in 1 week** vs $175 in 3 weeks |
| $165 in 1 week vs **$180 in 4 weeks** | **$165 in 1 week** vs $180 in 4 weeks |
| **$165 in 1 week** vs $195 in 7 weeks | **$165 in 1 week** vs $195 in 7 weeks |

Note: The options selected by the model are marked in bold font.

For the irrelevant model conditions, we created 11 pairs of objects, food or places similar in their function, valence and monetary values (see Supplementary Table 15). We told participants that the model decided between these objects based on her preference and that they should memorize these decisions.

Supplementary Table 15

*Study 4. Phase 2 offers in the control conditions: Decisions made by the irrelevant models*

| Irrelevant model / Low delay participants | Irrelevant model / High delay participants |
| --- | --- |
| roller skates vs **bike** | **bike** vs roller skates |
| **coffee** vs tea | tea vs **coffee** |
| roses vs **tulips** | **tulips** vs roses |
| baseball vs **football** | **football** vs baseball |
| bananes vs **peaches** | **peaches** vs bananas |
| jam vs **honey** | **honey** vs jam |
| **tablet** vs laptop | laptop vs **tablet** |
| woods vs **parks** | **parks** vs woods |
| pool vs **sea** | **sea** vs pool |
| umbrella vs **raincoat** | **raincoat** vs umbrella |
| sunglasses vs **cap** | **cap** vs sunglasses |

Note: The options selected by the model are marked in bold font.

***Experimental manipulation*.** The instructions and information provided in the experimental and control conditions were the same as in Study 3. In both the relevant and irrelevant model conditions the messages coming from the supposedly experimental partner were of similar length (77 and 79 words respectively), they had a similar structure and were both written in a cheerful ton. However, we adapted the message of the control conditions to the new context. The message read as followed:

*“I wish you good luck with the task! I enjoyed it a lot. A shame that it was not real rewards! I must admit that it was kind of tough to choose between the different options. I didn’t expect that because usually I’m quite good at deciding quickly. Actually, I could use some of the presented objects at home. Maybe I will stop at the grocery store to buy a few of these later, if I have time.”*

***Bonus Study*.** The Bonus Study consisted of three items aimed at assessing whether participants had procrastinated to perform this study (“Before beginning the Bonus Study, I thought several times “I’ll do it later “, even though I could have done it right away”, “I felt that I might run out of time to complete the Bonus Study because I delayed doing it until the last minute”, “When I agreed to do the Bonus Study I planned to do it earlier, but I kept on postponing it for no good reason”; Cronbach’s α = .77). Participants answered these items on a 7-point scale (1: strongly disagree, 7: strongly agree).

**Results**

In the main text, we report separately the ANOVAs comparing the number of chosen delayed options at Phase 3 and at Phase 5 to test the hypothesis that modeling has an effect at both phases. Here we report the bigger single model including all the factors and all phases (2 Intertemporal choice styles: high delay vs low delay participants x 2 conditions: relevant vs irrelevant model x 3 phases: Phase 1 vs Phase 3 vs Phase 5). This ANOVA leads to the same results as the separate analyses reported in the main text. Indeed, we found a three way interaction IC style x condition x phase (F(2, 148) = 3.952, p = .021, ηp² = .051). In addition, we found a main effect of intertemporal choice style (F(1, 74) = 130.94, p < .001, ηp² = .639), participants classified as high delay participants in Phase 1 delaying overall more than those classified as low delay participants. Finally, we found a significant 2-way interaction between participants’ intertemporal choice style and the experimental condition to which they had been assigned (F(1, 74) = 12.545, p < .001, ηp² = .145) and a significant 2-way interaction between participants’ intertemporal choice style and the phase at which they were tested (F(2, 148) = 10.097, p < .001, ηp² = .120). All other main effects and interactions were non-significant (all Fs ≤ 0.32, p ≥ .74, ηp² ≤ .004).

**Part 1**. In addition to the three-way interaction reported in the main article, the 2 (between: low vs. high delay participants) × 2 (between: relevant vs. irrelevant model) × 2 (within; pre- vs post-exposure to the model) repeated measure ANOVA on participants’ intertemporal choices at Phase 1 and Phase 3 revealed several main effect and interactions (see Supplementary Figure 4). We found a main effect of participants’ IC style, low delayers delaying less than high delayers (F(1, 366) = 788.39, *p* < .001, η*p*² = .68). We also found that participants’ IC style significantly interacted with the model relevance (F(1, 366) = 15.76, *p* < .001, η*p*² = .04) and the exposure to the model (F(1, 366 = 59.23*, p* < .001, η*p*² = .14).


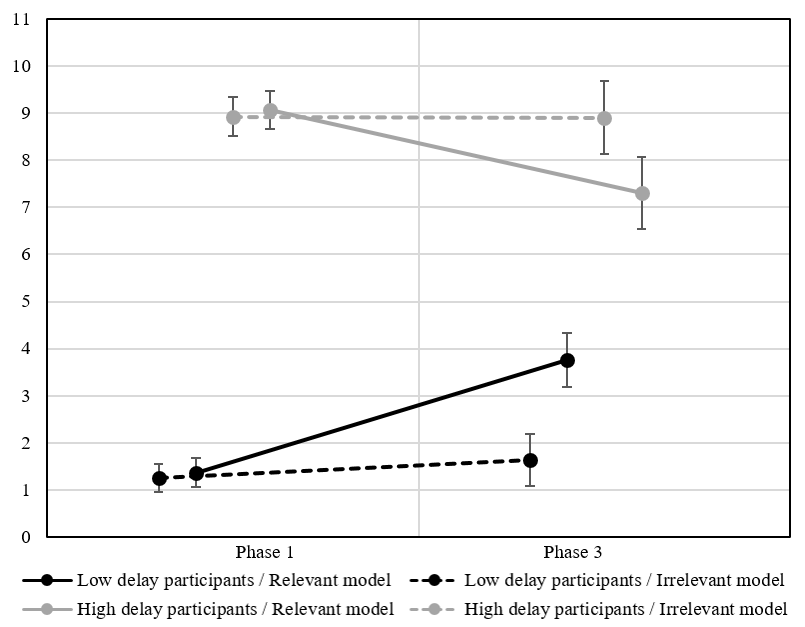
Supplementary Figure 4. Study 4. Mean delayed choices before (Phase 1) and after exposure (Phase 3) (N = 370). Error bars represent 95% between-subjects CIs.

**Part 2.** The descriptive statistics for Part 2 are presented in Supplementary Table 16.

Supplementary Table 16

*Study 4. Descriptive statistics for Part 2.*

|  | Relevant model | | Irrelevant model | |
| --- | --- | --- | --- | --- |
|  | High delay participant | Low delay participant | High delay participant | Low delay participant |
| Return frequency to the Bonus Study  (Percentage of returns among the participants who declared wanting to participate in the Bonus Study) | 51  (79.69 %) | 66  (66%) | 50  (80.65%) | 73  (66.97%) |
| Mean procrastination score (SD) | 2.00 (1.40) | 2.15 (1.47) | 2.02 (1.30) | 1.90 (1.23) |

*Time to return to the Bonus Study.* One could argue that participants who exhibit a tendency to procrastinate may return to the Bonus study later. The experiment was not designed to test this hypothesis as we had instructed participants to perform the Bonus study at any point in time between 1 and 49 hours after Part 1. Thus, it was not more accurate for participants to return earlier; they may have had other commitments that prevented them to perform the Bonus Study sooner. Still the reader may wonder whether return time was affected by our main factors. To answer this question, we performed a 2 (participants’ intertemporal choice style) × 2 (Model relevance) ANOVA. Neither the main effects nor the interaction led to any significant result (Intertemporal Choice Style: F(1, 253) = 0.027, p = .869, ηp² < .001; Model: F(1, 253) = 0.004, p = .950, ηp² < .001; Intertemporal Choice Style × Model: F(1, 253) = 0.660, p = .417, ηp² = .003).

**Part 3**. Like with Part 1 results, in Part 3, in addition to the three-way interaction reported in the main article, the 2 (between: low vs. high delay participants) × 2 (between: relevant vs. irrelevant model) × 2 (within; pre- vs post-exposure to the model) repeated measure ANOVA on participants’ intertemporal choices at Phase 1 and Phase 5 revealed several main effect and interactions. We found a main effect of participants’ IC style, low delayers delaying less than high delayers (F(1, 74) = 153.37, *p* < .001, η*p*² = .68). We also found that participants’ IC style significantly interacted with the model relevance (F(1, 74) = 7.78, *p* = .007, η*p*² = .10) and the exposure to the model (F(1, 74 = 16.49, *p* < .001, η*p*² = .18).

**References**

Grafton, S. T. (2009). Embodied cognition and the simulation of action to understand others. *Annals of the New York Academy of Sciences*, *1156*, 97–117.

1. The Cronbach’s αs reported here were calculated on Study 3 data. [↑](#footnote-ref-1)
